# Bloom Syndrome Presenting With Early‐Onset Myelodysplastic Syndrome and Triple Overlapping Vascular Neurocutaneous Phenotypes: A Case Report

**DOI:** 10.1002/ccr3.73007

**Published:** 2026-06-28

**Authors:** Elham Shahgholi, Nahid Khosroshahi, Sara Sadeghi

**Affiliations:** ^1^ Department of Pediatric Hematology‐Oncology Bahrami Children's Hospital, School of Medicine, Tehran University of Medical Sciences Tehran Iran; ^2^ Department of Pediatric Neurology Bahrami Children's Hospital, School of Medicine, Tehran University of Medical Sciences Tehran Iran; ^3^ Pediatric Growth and Development Research Center, Institute of Endocrinology Iran University of Medical Sciences Tehran Iran

**Keywords:** Bloom syndrome, genomic instability, monosomy 7, myelodysplastic syndrome, neurocutaneous overlap

## Abstract

Bloom syndrome is a rare autosomal recessive chromosomal instability disorder characterized by growth deficiency and early‐onset malignancies, and its coexistence with multiple vascular neurocutaneous syndromes is exceptionally uncommon. We report an 8‐year‐old girl who presented with severe growth failure and persistent pancytopenia. Bone marrow examination showed hypocellularity with monosomy 7, consistent with myelodysplastic neoplasm, and molecular analysis identified a homozygous pathogenic BLM variant (NM_000057.4:c.796C>T; p.Arg266Ter). Clinically, she exhibited overlapping neurocutaneous features within the spectrum of Sturge–Weber syndrome, Klippel–Trénaunay syndrome, and phakomatosis pigmentovascularis. The course was complicated by severe sepsis, leading to death before hematopoietic stem cell transplantation could be performed. This case highlights the importance of considering inherited cancer predisposition syndromes in children with cytopenia, growth failure, and complex neurocutaneous phenotypes.

## Key Clinical Message

Bloom syndrome should be considered in children with growth failure, cytopenia, and chromosomal breakage mimicking Fanconi anemia, particularly when overlapping neurocutaneous features prompt germline evaluation. Profound sensitivity to radiation and genotoxic agents necessitates strict avoidance of excessive exposure and reduced‐intensity conditioning for hematopoietic stem cell transplantation, the only curative treatment.

## 1 Introduction

Bloom syndrome (BS) is a rare autosomal recessive chromosomal instability disorder caused by pathogenic variants in the BLM gene on chromosome 15q26.1, encoding a RecQ helicase essential for DNA replication and genomic maintenance. Defective BLM function results in increased sister chromatid exchange and chromosomal breakage [1, 2].

BS is classically characterized by severe growth deficiency, sun‐sensitivity, characteristic long, narrow face, erythematous rash and a markedly increased risk of early‐onset malignancies [1, 2, 3].

Myelodysplastic syndrome (MDS) represents a recognized early hematologic manifestation of BS. Among the reported cytogenetic abnormalities, monosomy 7 is one of the most frequent and is associated with a high risk of progression to acute myeloid leukemia [3, 4, 5].

Neurocutaneous disorders or phakomatoses, comprise a heterogeneous group of conditions affecting the central nervous system and skin. Vascular neurocutaneous syndromes including Sturge–Weber syndrome (SWS), Klippel–Trénaunay syndrome (KTS), and phakomatosis pigmentovascularis (PPV) are characterized by capillary malformations, vascular anomalies, and pigmentary lesions with potential neurologic and ophthalmologic involvement [6, 7, 8]. Overlapping neurocutaneous phenotypes, in which features of more than one defined syndrome coexist, are thought to result from complex postzygotic somatic mosaicism [9].

We report a patient with molecularly confirmed BS and monosomy 7–associated MDS who also exhibited triple overlapping vascular neurocutaneous features involving SWS, KTS, and PPV, illustrating the diagnostic challenges that may arise at the intersection of constitutional genomic instability and mosaic developmental disorders.

## 2 Case Presentation

### 2.1 Patient Information

An 8‐year‐old girl, the second child of consanguineous parents with one healthy sibling, was referred for evaluation of persistent pancytopenia. She was born at term following intrauterine growth restriction. There was no family history of hematologic malignancy or known genetic disorders.

At birth, a unilateral facial port‐wine stain and congenital glaucoma were identified, and glaucoma surgery was performed during infancy. At 22 months of age, she developed generalized tonic–clonic seizures and subsequently exhibited global developmental delay. Her growth parameters remained below the 3rd percentile for age.

### 2.2 Clinical Findings

Physical examination revealed severe growth retardation and a long, narrow face with malar hypoplasia and micrognathia (Figure 1a,b).

**FIGURE 1:**
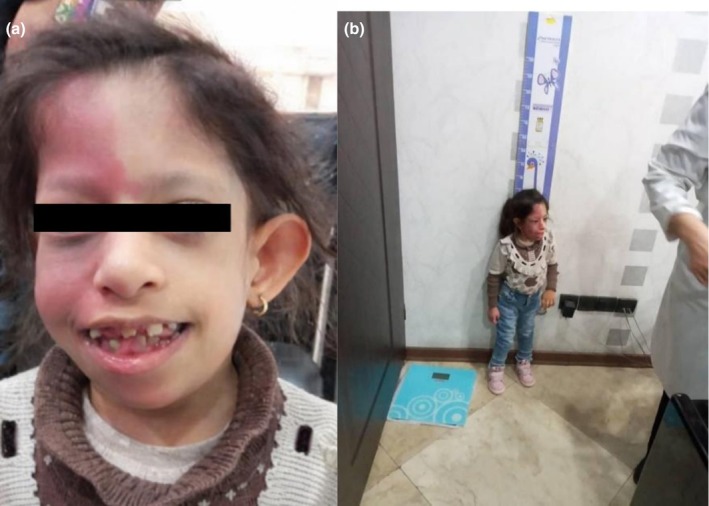
(a, b) Facial appearance and severe growth failure characteristic of Bloom syndrome. (a) Long, narrow face with malar hypoplasia and micrognathia. (b) Marked short stature consistent with severe growth failure.

Cutaneous findings included a unilateral right‐sided facial capillary malformation involving the ophthalmic and maxillary divisions of the trigeminal nerve, ocular melanosis consistent with nevus of Ota, extensive dermal melanocytosis over the trunk and back, and asymmetric hypertrophy of the right upper limb with associated vascular abnormalities (Figure 2a–d).

**FIGURE 2:**
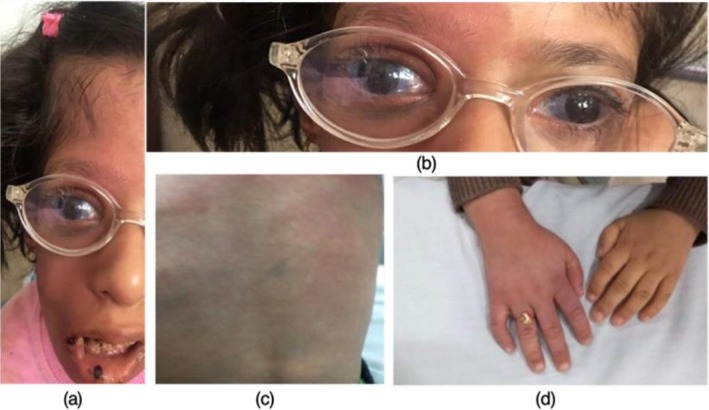
(a) Unilateral facial capillary malformation (port‐wine stain) involving the right V1–V3 trigeminal dermatomes, associated with congenital glaucoma, findings consistent with SWS; (b) Ocular melanosis (nevus of Ota), representing the pigmentary component of PPV; (c) Extensive dermal melanocytosis over the back and abdomen, further supporting classification within the spectrum of PPV; (d) Marked hypertrophy of the right hand with soft tissue and bony overgrowth, a characteristic feature of KTS.

Based on clinical findings, the patient demonstrated clinical features consistent with the spectrum of Sturge–Weber syndrome, Klippel–Trénaunay phenotype, and phakomatosis pigmentovascularis, constituting a triple overlapping vascular neurocutaneous phenotype (Figure 3). For Sturge–Weber syndrome, facial port‐wine stain and congenital glaucoma satisfied two of three cardinal criteria; however, definitive leptomeningeal angiomatosis was not demonstrated on gadolinium‐enhanced MRI. For Klippel–Trénaunay syndrome, the patient exhibited two of the three cardinal features; segmental capillary malformation (port wine stain) and asymmetric limb hypertrophy, while venous/lymphatic malformation was not identified. Two of three cardinal diagnostic features supported a Klippel–Trénaunay phenotype in this patient. For phakomatosis pigmentovascularis, extensive dermal melanocytosis and ocular nevus of Ota in conjunction with capillary malformation supported classification within the PPV spectrum. As somatic molecular testing of vascular lesions was not feasible, these diagnoses are presented as clinical phenotypes rather than molecularly confirmed entities. No hepatosplenomegaly or lymphadenopathy was detected.

**FIGURE 3:**
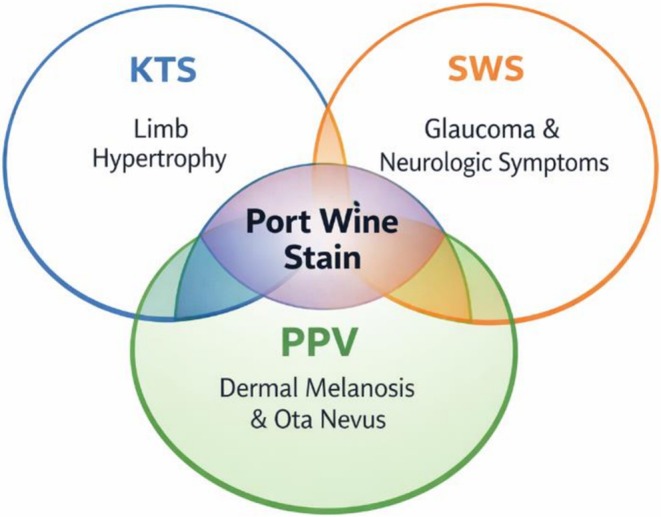
Diagram illustrating triple overlap of SWS, KTS, and PPV, with shared port‐wine stain and syndrome‐specific features.

## 3 Diagnostic Assessment

### 3.1 Neuroimaging

Brain magnetic resonance imaging demonstrated right hemispheric cortical atrophy, predominantly involving the occipital region (Figure 4).

**FIGURE 4:**
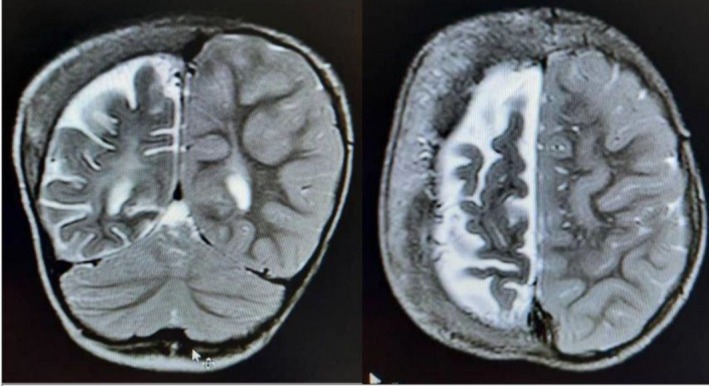
Brain MRI showing right hemispheric cortical atrophy. Axial T2‐weighted brain MRI demonstrates prominent right hemispheric cortical atrophy with ex‐vacuo enlargement of the extra‐axial CSF spaces, without evidence of a compressive subdural collection or active midline shift. No leptomeningeal enhancement was identified on gadolinium‐enhanced sequences.

### 3.2 Laboratory Evaluation

Laboratory evaluation revealed persistent pancytopenia with macrocytosis. Bone marrow examination showed hypocellularity for age with trilineage hematopoiesis and no excess blasts (< 5%). Flow cytometry did not reveal evidence of acute leukemia.

### 3.3 Cytogenetic Analysis

Cytogenetic analysis of bone marrow cells demonstrated monosomy 7 (Figure 5). In the presence of persistent cytopenia and a clonal cytogenetic abnormality, the findings were consistent with myelodysplastic neoplasm with monosomy 7 according to the 2022 World Health Organization classification.

**FIGURE 5:**
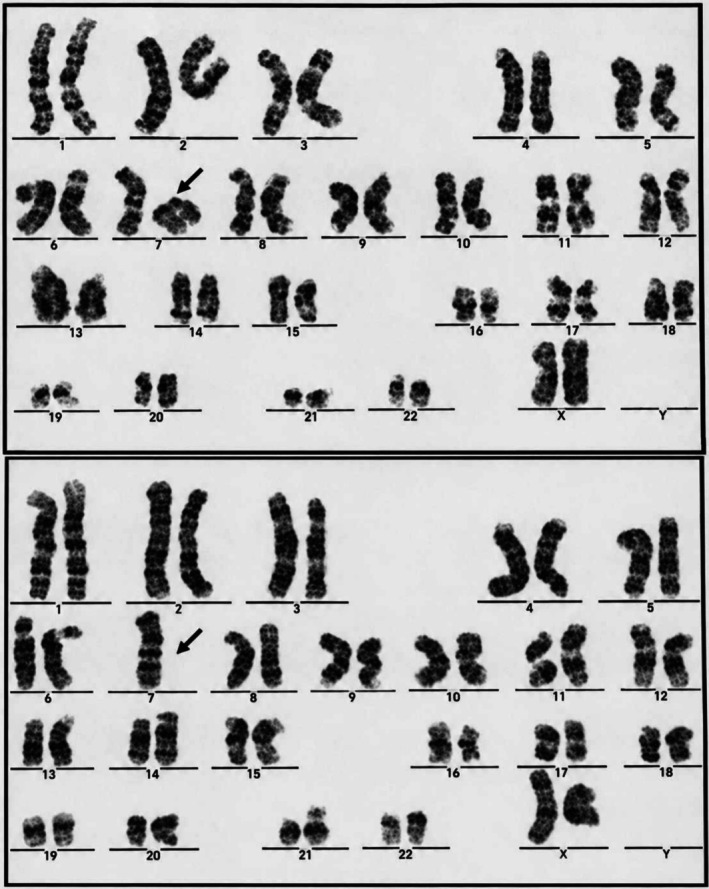
Conventional karyotype analysis of bone marrow cells (G‐banding, 550‐band resolution) demonstrating monosomy 7. Twenty metaphases were analyzed; 18 of 20 cells (90%) demonstrated monosomy 7, with a karyotype of 45,XX,−7.

Chromosomal breakage analysis using diepoxybutane (DEB) and mitomycin C (MMC) demonstrated increased chromosomal instability, initially raising suspicion for Fanconi anemia. The breakage rate was approximately 0.8 breaks per cell (normal < 0.1 breaks/cell) (Figure 6). Sister chromatid exchange (SCE), The characteristic cytogenetic finding of Bloom syndrome was not detected in the patient's chromosomal analysis, as specific BrdU‐based differential staining for SCE evaluation was not performed.

**FIGURE 6:**
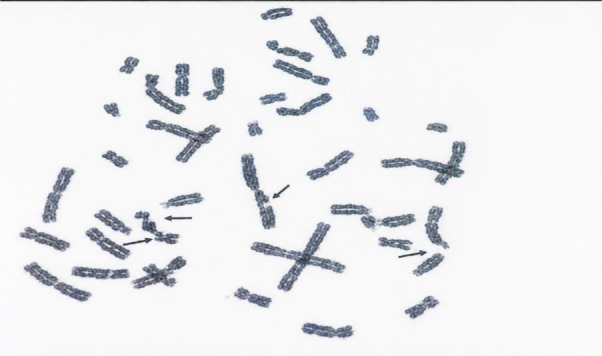
Metaphase spread demonstrating DEB/MMC‐induced chromosomal breaks and radial configurations (arrows). The marked chromosomal instability initially suggested Fanconi anemia before molecular studies confirmed Bloom syndrome.

However, the presence of severe growth retardation, dysmorphic facial features, and an overlapping neurocutaneous phenotype prompted further genetic investigations. Subsequent whole‐exome sequencing followed by Sanger confirmation established the diagnosis of Bloom Syndrome.

Sanger sequencing identified a homozygous nonsense variant (NM_000057.4:c.796C>T [p.Arg266Ter]), predicted to result in truncation of the encoded RecQ helicase protein. The variant was classified as pathogenic according to ACMG criteria (PVS1, PM2). Segregation analysis confirmed heterozygous carrier status in both parents and the paternal grandfather, consistent with autosomal recessive inheritance (Figure 7).

**FIGURE 7:**
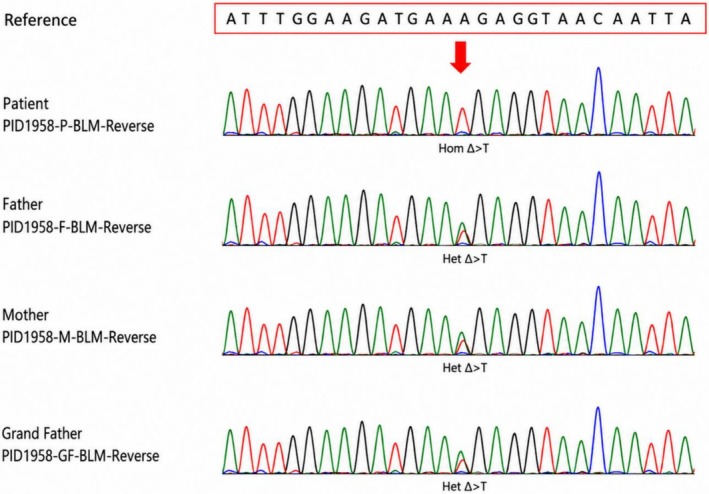
Sanger sequencing confirming a homozygous pathogenic variant (homozygous nonsense variant (NM_000057.4:c.796C>T [p.Arg266Ter])) in the BLM gene in the patient, with heterozygous carrier status in family members.

Because of severe hematologic instability, tissue biopsy and somatic mutation analysis of the neurocutaneous lesions could not be performed.

### 3.4 Therapeutic Intervention and Follow‐Up

The patient received intensive supportive care, including transfusions, broad‐spectrum antibiotics, antiepileptic therapy, and close multidisciplinary monitoring. Given the profound chromosomal instability and photosensitivity associated with Bloom syndrome, strict precautions were taken to minimize exposure to sunlight, ionizing radiation, and potentially genotoxic agents. Because of persistent pancytopenia and emerging monosomy 7 and MDS, urgent evaluation for allogeneic HSCT was initiated and reduced‐intensity conditioning (RIC) strategies were discussed due to anticipated toxicity with standard myeloablative regimens. Before definitive therapy could be performed, the patient developed fulminant sepsis complicated by disseminated intravascular coagulation and died despite supportive care.

## 4 Discussion

This case describes an exceptionally rare concurrence of molecularly confirmed Bloom syndrome (BS), very early‐onset myelodysplastic syndrome (MDS) with monosomy 7, and a triple overlapping vascular neurocutaneous phenotype. While each entity is uncommon individually, their coexistence in a single patient represents a diagnostically complex presentation.

In BS, intrinsic genomic instability underlies the increased risk of early onset malignancies, particularly hematologic disorders [1, 2]. MDS is a recognized early manifestation, and monosomy 7 has been reported in BS‐associated cases, conferring a high risk of leukemic transformation and adverse prognosis [3, 4, 5]. Registry data demonstrate a substantial lifetime cancer burden in BS, with cumulative malignancy incidence approaching 83% by age 40 and reduced median survival [10]. In addition, heightened sensitivity to DNA damaging agents due to defective DNA repair complicates therapeutic management and necessitates cautious treatment strategies [2].

Concomitantly, our patient demonstrated clinical features consistent with the spectrum of three vascular neurocutaneous syndromes. Sturge–Weber syndrome (SWS) is supported clinically by involvement of at least two of three domains: cutaneous capillary malformation, ocular abnormalities such as glaucoma, and leptomeningeal involvement [11, 12]. In our patient, facial port‐wine stain and congenital glaucoma satisfied two established criteria. Although seizures and hemispheric atrophy were present, definitive radiologic evidence of leptomeningeal angiomatosis was not demonstrated. Magnetic resonance imaging was preferred over computed tomography to minimize ionizing radiation exposure in the context of genomic instability.

Klippel–Trénaunay syndrome (KTS) is diagnosed clinically when at least two of three cardinal features capillary malformations, venous anomalies, and limb hypertrophy are present [13]. In this patient, the coexistence of segmental capillary malformation and asymmetric limb hypertrophy fulfilled these criteria.

Phakomatosis pigmentovascularis (PPV) is characterized by the coexistence of vascular malformations and pigmentary abnormalities, most commonly dermal melanocytosis [7]. In our patient, the presence of extensive dermal melanocytosis and ophthalmic nevus of Ota in association with capillary malformations supported classification within the PPV spectrum.

In neurocutaneous syndromes, the segmental distribution of cutaneous findings supports a postzygotic mosaic mechanism. Activating GNAQ (and less commonly GNA11) variants have been identified in SWS [5, 13], while somatic PIK3CA variants are most frequently implicated in KTS [7, 14]. Mosaic genetic alterations have also been reported in PPV [8, 14]. Although tissue‐based molecular confirmation was not feasible in this case, molecular testing is not mandatory for clinical diagnosis, and the absence of genetic confirmation does not preclude accurate clinical classification [14].

Overlap neurocutaneous syndromes refer to the coexistence of clinical features from two or more distinct neurocutaneous disorders within the same patient, reflecting shared developmental and mosaic pathogenic mechanisms. Rare triple‐overlap neurocutaneous phenotypes have been described and are generally interpreted as manifestations of complex postzygotic mosaicism [9, 15].

The coexistence of overlapping neurocutaneous syndromes with Bloom syndrome in this patient may be explained by three possible mechanisms: postzygotic somatic mosaic mutations facilitated by Bloom syndrome–associated genomic instability, a coincidental coexistence of independent disorders, or a previously unrecognized expansion of the Bloom syndrome phenotypic spectrum.

No direct pathogenetic link can be established in the present case, and somatic testing of vascular tissue would have been required to address this question definitively.

In patients with Bloom syndrome, underlying DNA repair defects increase susceptibility to severe toxicity from conventional cytotoxic therapy. In cases complicated by Myelodysplastic syndrome with monosomy 7, hematopoietic stem cell transplantation remains the only potentially curative treatment, although reduced‐intensity conditioning regimens may be required to minimize treatment‐related toxicity [16].

## 5 Conclusion

This case highlights a rare and diagnostically challenging presentation of Bloom syndrome associated with myelodysplastic syndrome and overlapping vascular neurocutaneous phenotypes. The combination of growth failure, persistent cytopenias, and mosaic cutaneous findings should raise suspicion for underlying chromosomal instability syndromes and prompt comprehensive genomic evaluation, including consideration of somatic mosaic mutations in complex phenotypes. Early diagnosis is essential because patients may exhibit increased sensitivity to genotoxic therapies and ionizing radiation, and timely recognition may guide surveillance, treatment modification, and consideration of hematopoietic stem cell transplantation as a potentially curative approach.

## Author Contributions

**Elham Shahgholi:** conceptualization, data curation, formal analysis, investigation, methodology, project administration, resources, supervision, validation, visualization, writing – original draft, writing – review and editing. **Nahid Khosroshahi:** data curation, investigation, validation, writing – review and editing. **Sara Sadeghi:** data curation, investigation, resources, visualization, writing – original draft, writing – review and editing.

## Funding

The authors have nothing to report.

## Ethics Statement

The authors have nothing to report.

## Consent

Written informed consent was obtained from the patient for the publication of this case report.

## Conflicts of Interest

The authors declare no conflicts of interest.

## Data Availability

The data used in this article are available upon request from the authors.
